# Ethical considerations and challenges of sex education for adolescents in Iran: a qualitative study

**DOI:** 10.18502/jmehm.v13i2.2664

**Published:** 2020-04-11

**Authors:** Kobra Joodaki, Saharnaz Nedjat, Marziyeh Vahid Dastjerdi, Bagher Larijani

**Affiliations:** 1 *PhD Candidate in Medical Ethics, Department of Medical Ethics, School of Medicine,* *Tehran University of Medical Sciences, Tehran, Iran.*; 2 *Professor, Department of Epidemiology and Biostatistics, Knowledge Utilization Research Center,* *School of Public Health, Tehran University of Medical Sciences, Tehran, Iran.*; 3 *Associated Professor, Department of Obstetrics and Gynecology, School of Medicine, Tehran University of Medical Sciences, Tehran, Iran.*; 4 *Professor, Endocrinology and Metabolism Research Center, Endocrinology and Metabolism Clinical Sciences Institute, Tehran University of Medical Sciences, Tehran, Iran.*

**Keywords:** Ethical considerations, Sex education, Adolescent, Medical ethics, Reproductive ethics

## Abstract

Adolescence is a period in one’s lifetime during which sexual maturation occurs. Major changes and increased sexual instinct raise many questions in the minds of adolescents. Receiving wrong education or inappropriate information can affect adolescents’ life and future deeply. Obviously, ethical considerations cannot be ignored in nationwide macro policies and educational programs on such a sensitive issue. In this qualitative study, we attempted to explore the ethical considerations and challenges of sex education for adolescents.

The study was conducted between May 2015 and March 2017. Data were collected through semi-structured in-depth interviews with 25 participants, and MAXQDA 11 was used for coding.

Six hundred sixty-two codes (662) were extracted and classified into four categories: 1) the potential risks of sex education for adolescents; 2) the advantages of sex education for adolescents, and the approaches; 3) the challenges in the interval between sexual maturation and marriage, and the role of religion; and 4) the measures implemented in Iran.

Shame, embarrassment, and some cultural beliefs surrounding the subject of sex education are obstacles to providing adolescents with the necessary information. According to the principles of medical ethics, the main principle in sex education is beneficence, and sometimes infringement of confidentiality has its advantages.

## Introduction

Adolescence is a transitional period in life when rapid physical growth and development and sexual maturation lead to one’s physical capability to reproduce. Based on the definition offered by WHO, adolescents are “young people between the ages of 10 and 19 years” (1). Since adolescents and young adults are going through the growth stage, their health can be affected either positively or negatively by their interactions with their environment, their friends and peers, their parents and family members, people in the society, policies and social rules (2, 3). In the course of sexual maturation, adolescents undergo fundamental changes and face with questions and ambiguities, and if they do not receive the right answers, they turn to their peers or media such as satellite TV, Internet and cyberspace. In this way, they often do not find proper answers and may even receive wrong ones, which will be problematic in the future. Therefore, education on the subject of sex and sex ethics is a necessity that must not be overlooked, and not only has to be provided by the parents and the society, but is also a fundamental right of adolescents (4). Due to its unique characteristics, sex education is accompanied by a number of challenges in all countries and cultures, and unique ethical considerations are associated with it (5). Talking about these subjects in public is tabooed in some countries (6). In some Western countries such as England, having sexual activity is accepted as a norm for young adults, but even in those countries there is a tendency not to talk about sexual intercourse and contraception with adolescents (7). In many Muslim countries such as Iran, sexual intercourse outside of marriage is prohibited both culturally and religiously (8,9). In such countries, sex education is insufficient and restricted, and is often avoided by teachers since they do not feel right talking about such subjects (10) There is currently no ongoing policy on sex education in Iran.

Although sex education has improved and different aspects of it have been dealt with, there has been little discussion about the associated ethical issues or the development of moral codes (11). Many studies have explored education about puberty and sexual matters in Iran and have examined the necessity of sex education, the content of the educational material, educators, and time of the training rather than the existing challenges (8, 9, 12, 13, 14, 15). However, no studies have been conducted on the ethical considerations and challenges related to this topic thus far. There have been a few studies on the issue in Western countries that have dealt with the ethics of sex education in general rather than adolescents and the issues specific to them (16,17). Those studies that have included young adults were mainly focused on criticizing the policies and regulations of Western countries in either advocating abstinence or offering comprehensive sex education, and compared the outcomes of the two systems of education with each other; in other words, they have not often dealt with the ethical considerations surrounding sex education (18, 19).

It is clear that ethical considerations cannot be ignored when nationwide macro-policies and educational programs are involved, especially regarding such a sensitive issue. In this qualitative study, we tried to clarify the ethical considerations and challenges of sex education for adolescents in Iran.

## Methods

This qualitative study was conducted between May 2015 and March 2017.

We used in-depth interviews and sampling of special or unique cases in this qualitative study due to its being flexible and in-depth (20). Participants were selected purposefully, and the attempt was to choose diverse subjects who were knowledgeable and experienced in the topic of the research. To ensure confidentiality, their job and social status were not disclosed. The interviews lasted 25 - 65 minutes, the average duration being 42 minutes. The questions were open-ended and exploratory to encourage participants to express their personal experiences and help to conduct in-depth interviews. The interviews started with an explanation of adolescents’ access to cyberspace and an uncontrollable environment, and information was obtained directly or indirectly about the subject. The main questions were extracted from studies conducted on education about puberty and sexual subject matters in Iran, such as the necessity of sex education, the proper training age, choosing a suitable person for the purpose, teaching methods, educational content, the effect of cultural differences, the negative impact of knowledge or lack of education, and the difference between girls and boys. At the end of the interview, if the interviewees had adolescent offspring, they were asked: “What do you do for your adolescent offspring in the way of sex education?” The discussions continued until data saturation was reached when participants proposed no new items in the last three interviews. The interviews were transcribed verbatim. 

***Qualitative Analysis***

After a final review of the transcripts, the MAX-QDA 11 software was used for the coding process, and the data were analyzed through Corbin and Strauss’s content analysis method (21). The first stage or substantive coding was done, a theme was ascribed to basic concepts in sentences and paragraphs, and the main theme was identified. Subcategories also emerged when coding at the next stage, and similar themes were placed in one subcategory or group. The classified concepts were then compared with each other and subcategories were put in categories. At the final stage, the main categories were formed.

***The Trustworthiness of the Study***

The trustworthiness of the study was assessed to confirm data accuracy in terms of the following four criteria: dependability, credibility, transferability, and conformability (22). To increase the dependability of the research, the codes were reviewed several times in order to classify them and find the main themes and main categories. Knowledgeable participants were selected from a large variety of majors, and triangulation technique was used to increase the consistency of the study (23). The recorded files of some interviews, their transcriptions, and the extracted codes were sent to some of the participants to confirm the transcription and method of coding (conformability). To heighten the reliability of the research, an external rater was used. Five anonymous transcribed files were coded by a researcher who was not involved in the study. The similarity of coding was estimated to be more than 75 percent (transferability).

***Ethical Considerations***

The participants were informed about the purpose of the study and their verbal informed consent was obtained. The researcher guaranteed privacy and confidentiality of the information, protection of recorded voices, and anonymity of the transcriptions. Whenever asked by the participants, the researcher would turn the recorder off. It should be added that this article was part of a larger study done for a Ph.D. dissertation in medical ethics, approved by the Ethics Committee of Tehran University of Medical Sciences (Approval No. IR.TUMS.REC.1395.2508).

## Results

In the present study, twenty-six participants were interviewed. The demographic and background characteristics of the participants are presented in Table 1. After the interviews were transcribed, one of the participants asked to be excluded from the research.

**Table 1:** Demographic and background characteristics of participants (N=25)

Demographic	No.
Age (yr.) **25 - 44** **45 - 64** **65 and >**	10 11 4
Gender **Female** **Male**	16 9
Marital Status **Single** **Married**	5 20
Having Teenage Children **Yes** **no**	19 6
Education **Less than high school diploma** **High school diploma** **Bachelor’s degree ** **Master’s degree** **PhD** **Doctor of medicine degree**	1 1 4 2 7 10
Background Characteristics of Participants **Physicians and medical ethicists ** **Doctors of theology (Islamic studies) and religious experts ** **Clients in health centers, one with a primary school certificate and ** **one with a high school diploma (12 grades of schooling)** **Clinical psychologists holding master’s degrees who worked in ** **health centers as psychologists and counselors for behavioral ** **disorders and as mental health experts** **Midwives: three of them offered contraceptive services as health ** **care providers in a clinic, and one worked in a maternity hospital** **Medical specialists: One pediatrician; one occupational medicine ** **specialist, and medical ethics teacher; one psychiatrist, religious ** **expert, and medical ethics teacher; one pathologist, religious ** **expert, and medical ethics teacher; three gynecologists and ** **obstetricians ** **Experts (graduates) in basic medical sciences including: A PhD in ** **clinical pharmacy who is also a medical ethics teacher; a ** **nutritionist, PhD, and religious expert; a PhD in environmental ** **health; An MD, PhD in reproductive health; a PhD in community-** **oriented medicine and fellow of sexual behavior (sexology); a PhD ** **in epidemiology**	3 1 2 2 4 7 6

Six hundred and sixty-two (662) codes were extracted from 25 interviews. These codes were classified into 25 themes, 12 subcategories and four categories (Table 2). 

**Table 2:** Ethical considerations and challenges of sex education for adolescents

Categories	Subcategories	Themes
Potential risks of sex education for adolescents	Harmful effects of sex education on adolescents	Having sexual intercourse without fear of its consequences
		Encouraging them to practice sex
	Disapproval of sex education by the society and organizations	Unpreparedness of the society and the educational system
		Interference between cultural and religious affairs
Advantages of sex education for adolescents, and the approaches	Harms of not offering sex education to adolescents	Misconception that adolescents are encouraged to practice sexual acts by being informed
		Violation of adolescents’ rights
		Unwanted pregnancies
		Adolescent sexual abuse
		Disadvantages of not knowing about a healthy spousal relationship
		Getting wrong information from inappropriate resources
	Appropriate age for sex education	The necessity for sex education and providing the needed information
		Age-appropriate education
	Educators	The role of the family
		Friends and peers
		The role of cyberspace
		Teachers in schools
		Education by a specialist
		Media
		Pharmacies
	Methods of sex education for adolescents	Educational content
		The method for education
	Decreased age of starting sexual intercourse	Decreased age of learning about sex topics
		The role of technology advancement and modernity
		Increased sexual relationships in adolescence
Challenges in the interval between sexual maturation and marriage, and the role of religion	The necessity of decreasing the age of marriage	
	The necessity of establishing a moral institution to resolve the challenges pertaining to the interval between sexual maturity and social maturity	
	Abstinence	Creating normal anxiety to prevent sins
Measures implemented in Iran	Teaching about puberty in universities and schools	
	Lack of transparency in the education system of Iran	Inconsistencies related to sex education in schools

The four main categories included: the potential risks of sex education for adolescents; the advantages of sex education for adolescents, and the approaches; the challenges in the interval between sexual maturation and marriage, and the role of religion; and the measures implemented in Iran. Subcategories, themes, and some participants’ statements are presented in Tables 3 - 6. 

**Table 3:** Category of the potential risks of sex education for adolescents

Subcategories	Themes	Quotes and Examples
**Harmful ** **effects of sex ** **education for ** **adolescents**	Having sexual intercourse without fear of its consequences	One of the medical specialists stated: “*We teach them. We help a 13-year-old girl; an 11-year-old boy * *satisfies his sexual desire. We help them, we prepare the way and the * *ground for their sexual activities. They experience it whenever they * *can.*” (No. 24) Another medical specialist mentioned: “*The education is for times when sexual activities are legitimate and * *within the Islamic framework. Outside of this framework, adolescents * *should remain uninformed in order not to be sexually stimulated. * *Once the function of a body organ is known, especially in case of * *reproductive organs, they want to put it into action to see the effects. * *Simultaneously, their sexual desire is triggered. Considering all this, our * *educational measures help to propagate sexual immorality.*” (No. 21)
	Encouraging them to act	One of the medical ethicists maintained: “*Sex is a powerful force. It is neither the desire for something edible * *or drinkable, nor an artistic work or music, so we cannot expect an * *adolescent to control himself/herself if he/she craves it. Can he/she? * *Definitely not! If you offer him/her sex education, you have actually * *prepared the ground for him/her to have sexual activities; he/she does * *it in any possible way, the first opportunity he/she gets.*” (No. 16) On the subject of sex education and provoking curiosity in adolescents, one of the medical specialists stated: “*I cannot say this for sure, not without evidence, but based on my own * *experience, I think if sex education starts sooner than the appropriate * *time, or if it is done very openly, it provokes curiosity.*” (No. 7) One of the clients of a health center explained: “*Sometimes knowledge leads to wrongdoing; perhaps it depends on * *the kind of family the child is brought up in.*” (No. 4)
**Disapproval ** **of sex ** **education by ** **the society ** **and ** **organizations**	Unpreparedness of the society and the educational system	Highlighting the unpreparedness of the social climate, a Ph.D. believed that sex education puts young adults at risk: “*Naturally, the society is not ready yet so that we can offer sex * *education directly to adolescents. Why isn’t it ready? Because the * *educational system does not have sufficient platforms for this; we are * *always worrying about the challenges, and we should always be * *cautious not to jeopardize adolescents and put them at risk by giving * *them sex education.*” (No. 19)
	Interference between cultural and religious affairs	One of the medical specialists mentioned: “*We have a major challenge: the interference between religious and * *cultural views. Most of the times religion allows us but culture * *doesn’t. We do not have any rules about this. Based on our * *constitution, we borrowed a rule from religion, but even our religious * *leaders gave in to politics. This is the very thing that keeps us silent. * *Why should I victimize myself more in my domain of work where I am * *influential but not supported by the system?*” (No. 7)

**Table 4:** Category of the advantages of offering sex education to adolescents, and the approaches

Subcategories	Themes
**Harms of not offering sex ** **education to adolescents**	Misconception that adolescents are encouraged to practice sexual acts by being informed
	Violation of the rights of adolescents
	Unwanted pregnancies
	Adolescent sexual abuse
	Disadvantages of not knowing about a healthy spousal relationship
	Getting wrong information from inappropriate resources
**Appropriate age for sex education**	The necessity for sex education and giving needed information
	Age-appropriate education
**Educators**	The role of the family
	Friends and peers
	The role of cyberspace
	Teachers in schools
	Education by a specialist
	Media
	Pharmacies
**Methods of sex education for ** **adolescents**	Educational content
	The method for education
**Decreased age of starting sexual ** **intercourse**	Decreased age of learning about sex topics
	The role of technology advancement and modernity
	Increased sexual relationships in adolescence

**Table 4-1:** Subcategory of the harms of not teaching sex education to adolescents

**Themes**	**Quotes and Examples**
**Misconception that ** **adolescents are ** **encouraged to ** **practice sexual acts ** **by being informed**	One of the PhDs disagreed with the view that being informed of sexual subject matters encourages adolescents to have sexual relations: “*Because I am talking about an adolescent, a human being. Those who disagree with sex * *education were themselves once adolescents; they are human beings. Firstly, they have to * *challenge themselves, they should go deep into their mind. You, I, that physician, that * *religious leader should go deep into our minds and ask ourselves, ‘once I became familiar * *with sexual relations in my sexual world, did I practice sex*?*’ *” (No. 19)
**Violation of ** **adolescents’ rights **	One of the PhDs considered the necessity of education as one of the main rights of adolescents: “*Yes, they have a right to know. They have the right to know what the norms of their sexual * *value system are. They are not like weed that grows automatically. We as adults, we as * *knowers, I as the mother or teacher should know how to give value to their sexual world. * *When they understand this value, certainly their responsibility increases*.” (No.19)
**Unwanted ** **pregnancies**	One of the PhDs explained: “*I wonder what the authorities plan to do about what is currently happening and what we * *hear now and then. It seems they want to ignore these adolescents so that … this means we * *let young adults give birth to illegitimate children*.” (No. 25)
**Adolescent sexual ** **abuse**	One of the clinical psychologists stated: “*We, as psychologists, believe that we should make even pre-school and school children * *aware of the intimate parts of their bodies to prevent potential abuse. We believe that from * *4-5 year-old children and younger to adults who, for instance, are university students, all * *need to be given scientific and academic sex education under the supervision of a * *professional. There is no difference; everyone needs sex education their age. Children * *need to be educated due to the rapes that are reported more frequently nowadays*.” (No.12)
**Disadvantages of ** **not knowing about ** **a healthy spousal ** **relationship**	A PhD, who was also a religious expert, stated: “*An important problem is lack of knowledge that is essential for young couples. This * *shortage manifests itself in disagreements, misunderstandings separations, and other * *problems*.” (No. 23) A gynecologist and obstetrician explained: “*We have cases who have been married for a couple of years, but cannot bear the smallest * *sexual touch or physical contact because of the fears they had, caused by myths their * *family told them about sexual relationships*.” (No. 20)
**Getting wrong ** **information from ** **inappropriate ** **resources**	Many experts pointed out to the harms of getting wrong information from inappropriate resources. One religious expert said: “*It is absolutely wrong to keep silent, to keep our mouths shut, not to talk about sexual * *subject matters, to let them get information from the disturbed market outside and immoral * *sources while we are happy that they know nothing*.” (No. 23) One of the medical ethicists stated: “*It may be harmful if the education is inappropriate. For instance, porn movies are the * *worst kind of education. These movies show sexual violations and unrealistic or wrong sexual * *behaviors in a way that the adolescent child thinks they are good and healthy sexual * *relationship. These movies not only are not educational but are absolutely harmful*.” (No. 18)

**Table 4-2:** Subcategory of the appropriate age for sex education

**Themes**	**Quotes and Examples**
**The necessity for ** **sex education and ** **giving the needed ** **information**	One of the religious experts explained: *“Some say that we should talk to adolescents about sexual subject matters when * *they enter puberty. I do not have a good experience of this. I feel children should be * *told about sex topics gradually from childhood when they come to understand * *sexuality – for instance at 9 or 10 years old – and we should make them ready little * *by little; then we can continue by telling them if extra information and tips are * *needed*.” (No 22). One of the midwives said: “*Sex education should be started from a very young age. Since website contents are * *replete with inappropriate and uncontrolled information about sex, the sooner sex * *education starts, the better it is.*” (No 11).
**Age-appropriate ** **education**	A very large number of codes were pertinent to age-by-age and age-appropriate education. In this respect, one of the medical ethicists stated: “*I think education should be age-by-age and start from the onset of puberty*.” (No. 24) One of the clinical psychologists explained: *“Some adolescents may concentrate on their studies and some may not. Perhaps * *some adolescents would like to experience sexual activities earlier than others and * *therefore questions arise for them about sexual topics. These things really matter*.” (No.12)

**Table 4-3:** Subcategory of educators and the resources for gaining information

Themes		Quotes and Examples
**The role of the ** **family**	One of the clinical psychologists explained: *“Generally we say that parents should start * *sex education from childhood by teaching their children the correct names of genital * *organs as private parts of their bodies. Nevertheless, if the parents do not know how to * *do this, they have to be taught*.” (No.12) *One of the PhDs stated: * *“Parents should have a complete parenting education program. They should know how * *to teach and convey educational contents to their offspring*.” (No.19) One of the religious experts said: “*I would prefer a friendly teacher as a counselor in the school to tell students about such * *topics rather than myself; it is better that mothers do not tell them anything*.” (No.22) One of the clients of a health center said: “*People’s manners of speaking about such topics are very different. Someone may speak * *comfortably, openly, and extensively with no censorship. I myself prefer not to give all * *the details*.” (No. 6) One of the medical ethicists stated: *“Due to the embarrassment and shame that exist in our families – and many believe * *there is no reason for it – we cannot talk about such topics. Unfortunately, this is the * *case in our family; therefore, parents cannot act as counselors of sex education for their * *offspring, even though they are the safest and most empathetic people for their kids. * *Sadly, this is how things are in our society*.” (No.16) With regard to the difference between girls and boys, one of the clinical psychologists stated: ”* Based on what I know and studied and learned in the workshops, it is preferable that * *sex education be given by the same-sex parent of the adolescent from puberty on; for * *girls, sex education should start from their first menstrual period. At puberty, a series of * *changes happen to boys. A 15-16-year old boy may have night-time erections. As parents * *we should be able to answer the questions of these curious minds*.” (No.12)
**Friends and peers**	Most of the participants believed that the role of friends and peers in sex education is negative as they may convey wrong and harmful information. One of the health care experts said: *“But if they get the information from their friends, they may be encouraged to have * *sexual experiences*.” (No.12)
**The role of ** **cyberspace**	Most participants referred to the role of cyberspace in education about these affairs and highlighted its uncontrollability, wide accessibility, and negative effects on the education of adolescents. One of the PhDs stated: *“Previously, children’s access to such information was limited. They could only ask * *their friends. Now all of them have access to cyberspace on their cell phones. Therefore, * *they certainly go there and get wrong information*.” (No.1)
**Teachers in schools**	One of the medical specialists stated: *“Such subjects should be taught by a teacher who is always present in the school, not by * *someone from outside who teaches and leaves. The reason is that a student may have no * *questions when he/she is being taught, but that does not necessarily mean that he/she has * *no problem or will not have any problems in the next week. Students should know that * *they can refer to the teacher who has taught them whenever they have a question. Of * *course, the teacher should be selected very carefully and cautiously*.” (No.8)
**Education by a ** **specialist**	One of the psychologists said: *“I emphasize that it should be a reliable form of education under the supervision of * *specialists. In this way, they are not sexually stimulated*.” (No.12) One of the PhDs explained: *“Iatrogenic factors that happen in case teachers are non-professional or non-expert in * *sex education are definitely harmful.*” (No.19)
**Media**	Participants advocated making use of the media for sex education, though cautiously. One of the medical ethicists stated: “*Education has to be started in the family; media should have a role in education too; * *the significant subject matters should be presented in mass educational media*.” (No.13)
**Pharmacies**	Due to adolescents’ referral to pharmacies to get contraceptives, one of the PhDs suggested the opportunity for sex education in pharmacies and said: *“If a 14-15-year old * *adolescent refers to a drugstore and asks for contraceptives, he/she can receive it. * *However, firstly he/she has to be guided. Here the adolescent is showing that he/she has * *the right to know. The pharmacist has to give the necessary information to him/her and * *guide him/her toward safe behaviors*.” (No.3)

**Table 4-4:** Subcategory of the methods of sex education for adolescents

Themes	Quotes and Examples
**Educational ** **content **	Teaching reliable scientific materials was highlighted by some participants. A clinical psychologist said: “*I agree with sex education 100 percent, but the content to be taught should be * *reliable and offered under the supervision of specialists*.” (No.12) Considering adolescents’ background information was also highlighted by many participants. One of the gynecologists stated: “*It’s almost a crime to cause adolescents to have an unhealthy attitude toward * *sexual subject matters; or distract the thoughts of adolescents who are only busy * *with their studies to issues which they were not supposed to know*.” (No.20) One of the medical ethicists explained: “*Some factors are influential here, for example, how much information they get * *from other resources. Because when education is wrong, they may receive wrong * *information*.” (No.18) Another question was, “Should adolescents’ living place and local culture influence the type and method of education?” Some of the participants highlighted the cultural differences between cities or even between various areas within a city, which lead to different needs of the adolescents for education. One of the clinical psychologists said: “*Education should be given based on adolescents’ continuum of development; it * *should be appropriate for their age range and in accordance with their culture * *and the family in which they are growing up*.” (No.12) One of medical ethicists said: “*There should be a specialized program for education based on adolescents’ age, * *gender, geographical location, culture, and the content of the subject matter to be * *taught. For instance, teaching some topics may have priority over some other * *topics when you are teaching boys; the same is true for education of some topics * *in accordance with age. Or education may be different based on whether you are * *teaching in a city or a village; it has to be. Seemingly we fail if we cannot develop * *a specialized program for education*.” (No.16)
**The method for ** **education**	Participants insisted on indirect teaching. One of the religious experts said: “*We are facing a dilemma: should we be silent and allow adolescents to do as * *they wish, or should we teach them responsibly and within a framework of * *educational and moral structure? But this doesn’t have to be announced explicitly * *on media or in public where 6-year-old children would hear and ask what the * *whole thing means. The information has to be given wherever it has to*.” (No.23) One of the midwives said: “*Yes, it should not be taught overtly. For instance, we can say to them that there * *are methods people use to prevent unwanted pregnancies; of course, this should * *be done as indirectly as possible; However, naming the methods may be * *inappropriate in some cultures; for instance, telling boys directly to use condoms * *may be very unpleasant. I myself have a son, it will be very embarrassing for me * *if someone comes and says this to my son*.” (No.14)

**Table 4-5:** Subcategory of the decreased age of starting sexual intercourse

Themes	Quotes and Examples
**Decreased age of ** **learning about ** **sex topics**	Most participants expressed their worries about the decreased age of learning about sexual topics. One health care expert who had been to a middle school to offer health education to students stated: “*They knew many things, I mean they knew much more than a 40-year-old woman in * *the past generation, they knew everything. I was supposed to teach them, but they * *themselves knew everything in detail*.” (No.10) Another health care expert told about students in the first grade of high school: “*I realized from what they said that they knew many things about sexual subject * *matters, they knew everything, and most of them stated this overtly*.” (No.14)
**The role of ** **technological ** **advancements ** **and modernity**	With respect to technological advancements and modernity, one of the medical ethicists explained: “*If you live in a city where you have electricity, a cell phone, and a tablet, you * *cannot restrict their accessibility to the resources*.” (No.18)

**Table 5:** Category of the challenges in the interval between sexual maturation and marriage, and the role of religion

Subcategories	Themes	Quotes and Examples
**The necessity to ** **decrease the age of ** **marriage**		One of the medical ethicists said: “*In a religious society, it can be helpful if the education is based * *mainly on informing adolescents, which leads to abstinence, and * *simultaneously the policies are to decrease the age of marriage. It is * *not reasonable to demand that individuals practice abstinence when a * *very strong force from outside is telling them the reverse. But if you * *focus your attempts on decreasing the age of marriage to 19 or 20 * *years, you can expect them to wait because in this way they think that * *they can marry 2 or 3 years later and tell themselves that when they * *finish high school, they will. In such conditions, you can expect * *abstinence from adolescents.*” (No.16)
**The necessity of ** **establishing a ** **moral institution to ** **resolve the ** **challenge of the ** **time between ** **sexual maturity ** **and social maturity**		One of the medical ethicists said: “*In the current situation, it is impossible. Therefore, the society can * *establish a moral institution in which adolescents can have sexual * *relationships in the interval between puberty and marriage; a * *relationship acknowledged by the society that commands commitment, * *not promiscuousness, and is based on common sense and ethical and * *social principles. Meanwhile, adolescents will receive sex education * *at the right time. Such an institution is absent in our society.*" (No.18)
** Abstinence**	Creating normal anxiety to prevent sins	The opinions about abstinence and adolescents’ ability to practice it were different. One of the medical ethicists stated: “*You cannot expect an individual whose sexual impulsions have been * *activated since 15 to practice abstinence. You don’t have the right to * *pressure him/her to wait until 30 to marry. This means that you want * *them to practice abstinence for half of their lives. Are the teachers * *who sex educate themselves able to do this?*” (No.16) One of the PhDs believed that intense sexual desire can be controlled through education: “*Why should young adults be taught to be animalistic or be told they * *are not able to control their sexual desires? If I am to teach them, I * *will tell them that sex drive exists, but it is controllable; I will tell * *them that sexual activities are not the only source of pleasure, we can * *derive pleasure from drinking this tea as much as the pleasure we * *derive from watching a woman or a man. Now they can choose for * *themselves. I will give them measuring devices to choose by. I will not * *speak nonsense. I will tell them to think and see which pleasure has * *priority for them, so they can choose*.” (No.19) One of the PhDs said: "*Such moral issues are not resolved by forbidding watching videos or * *satellite TV. These measures are not helpful. The only way is the way * *of the prophets. Prophets tried to prevent moral corruption by their * *power. If they had no power, they tried to train and guide human * *beings. How? By giving human dignity to human beings, through fear * *of divine retribution and promises of divine rewards*.” (No.23)

**Table 6:** Category of the measures implemented in Iran, and the related challenges

Subcategories	Themes	Quotes and Examples
**Teaching about ** **puberty in ** **universities and ** **schools**		One of the specialist physicians stated: “*At that time we did this: we set aside the educational system * *and financially supported any institution that had political * *power. We were friends with representatives in the Iranian * *parliament, and we knew university authorities. We went ahead * *and prepared puberty packages and gave them to the girls*.” (No.7) A Ph.D. said: “In *cooperation with the Ministry of Health, schools and the * *educational system can set certain objectives and develop a * *course for education in schools. Of course, this education * *should not violate the norms but should still be informing*.” (No.25)
**Lack of ** **regularity in ** **education in Iran**	Inconsistency of sex education in schools	In this regard, one of the Ph.Ds. said: “*What is happening today is that everyone acts as they wish. * *One school starts sex education very soon, another teaches all * *about it in detail, and another one does not offer any * *education, everyone does whatever they like*.” (No.9)

***1. The Potential Risks of Sex Education for Adolescents***

The participants disapproved of providing adolescents with sex education mainly on account of possible harmful effects. Some of the hazards pointed out by the participants were: engaging in sexual activity following sex education; having sexual intercourse without fear of the consequences; being sexually stimulated as a result of curiosity about sexual subjects; and feeling encouraged to experience sexual activities. The participants also emphasized the necessity for conducting a study on the role of sex education in propagating sexual relations. Furthermore, they strongly disagreed with open and direct sex education, reasoning that the society and the predominant climate of the Iranian educational system are still not ready for it. Most of the participants highlighted the taboo of talking about sex topics in the society and some of them mentioned the prevalent social atmosphere as the reason for their disagreement. 

This category was classified into two subcategories and four themes. The category and the related subcategories, themes, and some participants’ statements are presented in Table 3.

***2. The Advantages of Sex Education for Adolescents, and the Approaches***

In the current study, most of the participants agreed on the necessity of sex education. Although they mentioned the harms of lack of sex education, they indicated the dominant cultural atmosphere of the society and the taboo of talking about sexual subject matters in the society and families, and believed that public presentation of sex education can interfere with modesty and chastity in the society. Some of the participants did not see a formal and clear discussion about these subjects as appropriate and proposed certain considerations; for instance, they believed that sex education should not violate the norms or encourage an individual to have sexual activities. Most participants agreed on the importance of providing reliable materials for teaching and indirect education, and highlighted the necessity of educating individuals who have risky behaviors. 

In support of sex education, advocates mentioned the fundamental right of adolescents to be informed about the subject, the decreased age of learning about sexual matters and engaging in sexual relationships, the effect of advancements in technology and modernity on this matter, and the increase in unsafe sexual relationships among adolescents caused by the inappropriate behaviors of families, wrong education, and internet accessibility. As for the disadvantages of not offering sex education and using the *reductio ad absurdum* rule, they mentioned adolescent sexual abuse, receiving wrong information from inappropriate resources, increased rates of unwanted pregnancies, illegitimate births and abortion, lack of information about proper marital relations, increased divorce rates due to sex-related problems, and fear of building legal and legitimate sexual relationships. Some of the participants advocated sex education for adolescents, arguing that it would prevent unwanted pregnancies and thus decrease the rate of abortion and birth of illegitimate children.

This category was classified into five subcategories and 20 themes. The category is presented in Table 4, and its subcategories and related themes and some interviewees’ statements are separately shown in Tables 4-1, 4-2, 4-3, 4-4 and 4-5.

***3. The Challenges in the Interval between Sexual Maturation and Marriage, and the Role of Religion***

One of the challenges proposed by participants pertains to the interval that exists between puberty and marriage. The main concern was that it is not rational to educate adolescents on sexual relations and contraception but expect them to wait until they are married, which may be as long as 20 years away. 

This category was classified into three subcategories and one theme. The category and its related subcategories, themes, and some interviewees’ statements are presented in Table 5.

***4. The Measures Implemented in Iran,*** ***and the Challenges***

Most participants believed that there is no consistent policy regarding sex education in Iran, and the predominant atmosphere in the society and the Ministry of Education prevent the issue from being properly dealt with. They also considered the measures taken in Iran as insufficient. They believed that there is no clear policy with regard to sex education in schools, and that sex education is offered based on what the people in charge think should be done. Consequently, lots of students do not have sufficient awareness of pubertal health or the necessary skills to select proper sex behaviors.

This category was classified into two subcategories and one theme. The category and its related subcategories, themes, and some participants’ statements are presented in Table 6.

## Discussion

Like many other countries, there is no consistent policy on sex education in Iran, and the predominant atmosphere in the society is the main cause. Some of the arguments that have been proposed in opposition to sex education include the harmful effects of sex education on adolescents, non-existence of the required infrastructures in the educational system and schools, and the clash between culture and religion. However, sexual abuse of non-informed adolescents, and unwanted pregnancies and consequent unsafe abortions were pointed out as some of the harms and unpleasant outcomes of an absence of sex education. We will discuss our findings according to the different categories extracted in this qualitative study.

***The Potential Risks of Sex Education for Adolescents***

Only a few participants were against sex education for adolescents, and believed the appropriate time for sex education to be after puberty and at the time of marriage. The reason for their disagreement was the harms brought about by sex education. These results are in accord with those of other studies that showed most parents agreed with sex education for adolescents, and only a few were against it. In a survey, more than 80 percent of the parents agreed with sex education in schools and only a few parents disagreed because they were worried that sex education would provoke the curiosity of adolescents and encourage them to have sexual activities (24). In a nationally conducted study in the USA, the data obtained in surveys from parents of adolescents studying in high schools showed that 90 percent of parents believed that it is either absolutely or almost essential to offer sex education in schools. Only seven percent of the parents disagreed with sex education and providing information on contraception in schools. (19) Moreover, only a few studies reported that sex education caused an increase in sexual activities (25). 

***The Advantages of Sex Education for Adolescents, and the Approaches***

The findings of our study are in accordance with other studies conducted in Iran and elsewhere. Kalantary et al. reported that appropriate sex education leads to the development of adolescents’ self-esteem and their skills in decision-making as well as decreased high-risk behaviors (5). 

***The Harms of not Educating Adolescents in Sexual Matters***

Despite the common belief that sexual health education encourages adolescents to have sexual relations, a study shows that lack of sex education not only does not prevent sexual relationships, but also develops and solidifies false beliefs and information among Iranian adolescents. When adolescents do not receive sex education, they turn to porn journals and websites as well as sexual content on satellite TV channels (14). Also, a review of 65 articles showed 7.9 percent of the boys and 19.7 of the girls had been sexually abused under the age of 18 (26).

The above-mentioned information has been confirmed by numerous studies in which adolescents have expressed their desire for training in sexual matters. They believed that receiving detailed information at a younger age does not increase the chance of practicing sex, but limited knowledge may arouse curiosity and encourage adolescents to experience sexual relations (8). Lack of knowledge and the right attitudes toward pubertal and reproductive health may cause many adolescents to have negative feelings and a sense of guilt toward their pubertal processes and sexual desires, which may lead to consequent problems and adverse effects in their future married life (6). One study showed that receiving information about AIDS and sexual health correlated with more positive attitudes toward sexual abstinence. It also revealed that students’ needs and expectations in terms of learning about AIDS and sexual health are not satisfied in schools (18).

***Age-Appropriate Education***

One of the main challenges with regard to sex education was determining the proper time, and a consensus could not be achieved among participants in this respect. Most participants highlighted taking adolescents’ age and background information into account and opted for indirect education. Obviously, there is a different time for teaching various aspects of health (physical, sexual, mental and social) in puberty. Further investigations are needed to determine the proper time for education in each aspect of pubertal health, considering the sociocultural factors of the society (15). This education has to take place before the beginning of puberty, and the gender of the adolescent needs to be taken into account; in this way, adolescents become familiar with the signs and changes associated with puberty so that they will not be frightened by them, and will thus be able to resolve their problems (27). Informing adolescents of sex topics has to start a little sooner than the time they engage themselves actively in these issues (16). Most parents and teachers know the time for sex education, for instance issues such as fertilization of ovum and pregnancy may be discussed with girls near the time of marriage, and the best time for teaching about sexually transmitted diseases may be in high school. However, teachers consider the right time for sex education to be sooner than the time pointed out by parents (9). In another study on adolescents in Iran (2010), some students believed that education on topics such as menstruation is more helpful in the final years of primary school; they also commented that sex education at the time of marriage – which is common in Iran – is too late and senseless (28). In a review article it was mentioned that the proper time for teaching different aspects of pubertal health is different; according to this study, in order to bring about maximum benefits for adolescents, the proper time for sex education should be determined based on the sociocultural conditions of the society. This study pointed out that sex education for girls should be started before puberty and around the ages of 9 to 10 (8).

***Educators and Training Resources***

The first resource for education on puberty is families, especially mothers. Unfortunately, research findings show parents’ lack of sufficient information or skills for teaching valuable content to their offspring; moreover, feelings of shame and embarrassment lead to negligence in transferring experiences and knowledge by parents, or even in schools (5, 29, 30). Also, parents and educators sometimes do not have the necessary information about sexual behaviors and issues related to puberty. They may simply not know how to talk about such subject matters to adolescents (8, 31). In our study, participants named teachers as the most reliable resource for education after parents. However, they emphasized that teachers should have the required skills and proficiency on the subject. In most studies, adolescents reported that they received critical information about sexual subject matters from their friends, siblings and media rather than the material that is formally presented in schools. In a study, Malek et al. found out that adolescents’ resources for gaining information about sexual health are their close friends, photos, magazines, books, audiovisual tools, school teachings, physicians, Islamic religious leaders, counseling centers, family and close relatives, respectively (32). Studies also showed that receiving unreliable information from friends could be a risk factor for high-risk behaviors (33). Female adolescents’ primary resource of information about puberty is their mothers and near others in their family. Their resources about sexual subject matters, however, are their friends, which they confirmed to be unreliable (34, 35). Boys become familiar with sexual subject matters by their friends or in military service (29). Numerous studies have highlighted the negative role of satellite TV channels and the Internet, which they have shown to be not only unreliable resources for sex education, but also sexually stimulating (36, 37). Iranian adolescents are bombarded by multiple sexually stimulating messages that lack right and sufficient information about sexual reproduction. A study showed that learning about sex from peers and media correlates with increased probability of having sexual intercourse, while learning from adults (parents or religious leaders) will more likely lead to abstinence (38).

Another source of information that had not been reported by other studies was pharmacists. When adolescents refer to these professionals to obtain contraceptives, they can gain proper knowledge, learn how to protect themselves, and be warned of high-risk behaviors.

The other challenge reported by a few other studies (36, 38, 39) was the families’ awareness of sex education offered to their adolescent children. In our study, most participants emphasized that parents have to know the content of materials taught to their offspring and some of them highlighted that no education should be offered to the students without their parents’ permission; they also mentioned that informing the parents should be compulsory, especially when the students were in early adolescence years and under legal age. Mothers have been shown to have concerns and fears about their daughters’ vulnerabilities, and potential physical and psychological harm to them. 

There is a sort of conservatism in Iranian families pertinent to their offspring’s sex education. The discussion about such matters seems indecent to typical Iranian parents (39). A study showed Iranian adolescents and young adults are not sufficiently skilled to manage their sexual activities. The main reason for this problem was pointed out to be inappropriate interactions between parents and offspring (38). A study showed that mothers and fathers employ different methods when dealing with their children’s sexual issues; their approach to the subject is influenced by the sort of sex education that they themselves received, their value and belief system about sexual subject matters, and their attitudes toward such issues (36). 

A study by Sajjadi et al. showed that fathers’ awareness about puberty in boys is not at an acceptable level, and 88.9 percent did not have accurate information about the religious aspects of puberty in boys and the time they are obligated to take full responsibility under Islamic law (40). The results of another study which aimed to determine the educational needs of fathers for the puberty period of their sons showed that the highest frequency in the educational needs of boys during puberty is related to religious subject matters (41). Lack of fathers’ communicative skills is not exclusive to the Iranian society. For instance, mothers talk more than fathers about sexual subject matters to their sons in the US. Furthermore, girls are more comfortable talking about sex topics with their mothers than boys (42).

***Method for Sex Education***

The results of the current study are in line with those in other studies. Sex education should be offered in a way that it would cause the lowest rate of complications possible. Considering that all sexual subject matters are not at the same level in terms of being sexually stimulating, it is suggested that subject matters that are more so be educated indirectly (43).

Educational contents and materials have to be comprehensive, sufficient, and cover all the aspects of puberty. Furthermore, the method for teaching the materials must increase the effectiveness of education so that it will lead to skills development through proper planning and use of different methods (8). Training should not discourage individual from engaging in sexual practices, and the materials should cater to all personality types in adolescents. It is a widely held belief that that if sex awareness is not formed appropriately, it can be a risk factor for other problems. Moreover, sex education has to be proportionate to individuals’ gender, age, level of cognition, and sociocultural background (44). 

***Decreased Age of Starting Sexual Relations***

Most participants expressed their worries about the decreased age of learning about sexual topics. Considering the increase in sexual relationships among adolescents, participants highlighted the necessity of sex education, especially for individuals with high-risk behaviors, in order to improve their knowledge and prevent unpleasant outcomes. Some referred to the difference in the increased rate of adolescents’ sexual practices in various areas of the cities, and some who had experienced working in this field saw no such difference, but confirmed that the relationships were safer and more protected among families with higher educational levels.

Detailed statistics reported in studies of other countries showed that the above-mentioned factors were the main reasons for having approved laws for compulsory sex education in schools, but statistics are not accurate in Iran. However, according to the statistics published by the Iranian Ministry of Health and Medical Education and a study conducted by the Iranian Ministry of Education, the condition in Iran is similar to that in Western countries. One study confirms the increased rate of sexual relationships before marriage among Iranian adolescents (6, 45). Evidence shows that the pattern of HIV transmission due to sharing needles and drugs injection equipment has changed to unprotected sexual intercourse among Iranian young adults (46). In the report titled “Islamic Republic of Iran َ AIDS Progress Report: On Monitoring of the United Nations General Assembly Special Session on HIV and AIDS”, 6.1% of men and women aged between 19 and 24 answered they had had sexual activities under age 15 (47).

***The Challenges in the Time between Sexual Maturation and Marriage, and the Role of Religion***

One of the issues mentioned by some of the participants that had not been reported in any other study was about the relatively long time between sexual maturation and marriage. The sexual instinct is at a peak during this time, and there exists an abundance of environmental stimulation while adolescents have no prospect of marriage. The common sense among Iranians, the religion of Islam, and some Abrahamic religions recommends self-restraint (abstinence) in this period. Participants mentioned that it is not rational to expect one to practice abstinence sometimes 20 years after sexual maturation, considering the conditions in the society and the increased sexual stimulations in the community. Temporary marriage (sigheh) was also referred to as one solution to this problem. Moreover, the necessity to decrease the age of marriage was mentioned.

Studies confirm the effectiveness of religious beliefs and faiths on appropriate sex education and controlling and preventing sexual relationships before marriage or out of wedlock. In our research, religious experts and some other participants believed that Islamic doctrines are comprehensive in terms of sex education. They mentioned that sexual discipline in Islam starts from early childhood by giving gender identity to children. As children and adolescents develop, training should become more specific, deal with physical and psychological health, and highlight the legitimate use of sexual instinct. Education on puberty and sexual health is considered to be the duty of parents and is parallel to the educational role of prophets, God’s warnings and promises. They also believed that in addition to providing adolescents with the necessary information on sex topics, creating a fear of the unpleasant outcomes of sexual perversion is very useful. Sexual discipline in Islam means training individuals who can distinguish between halal (unforbidden) and haram (forbidden) when they reach puberty, recognize marital and spousal duties and responsibilities, refrain from unrestrained sexual relationships, and have virtues of Islamic modesty and chastity in their personality and manner of life (5). Another study showed that informing adolescents about sexual subject matters and important ethical values are the duty of parents from the Islamic perspective (34). A study indicated that religion has a significant role in refraining from high-risk behaviors. The possibility of establishing an illegitimate sexual relationship is 27 to 54 percent lower among religious young adults than unreligious ones. These adults also have fewer sexual partners compared to their peers (48). In a review article, Bahrami et al. confirmed the significant role of religion in individuals’ beliefs, especially in discouraging adolescents from having sexual relationships before marriage. This study also showed that the society is moving in the direction of sexual liberation, which endangers the Iranian culture and values. Besides, they stated that education has little role in postponing sexual behaviors until marriage and mentioned religious faith and spirituality as primary factors in preventing individuals from prohibited sexual relationships in Iran and other countries (49). Victor et al. studied 2202 adults in terms of the effect of moral perspectives on the number of sexual partners they had over the years (a sexual risk indicator). They concluded that moral worldviews influence sexual behaviors over time. However, they have a considerably protective effect only for individuals who have high levels of moral beliefs (50)

A study on the effect of the current policies regarding sex education in high schools of the United States on students’ risky sexual behaviors showed that all the policies advocating abstinence correlated positively with risky sexual behavior; comprehensive policies on sex education that offer a combination of contraception and abstinence, however, caused a decrease in the rate of risky sexual behaviors. This study concluded that policies or programs based on “abstinence-only” as a choice for adolescents are incomplete both scientifically and ethically (51).

***Measures Taken in Iran and the Related Challenges***

Most participants believed that on the one hand, there is no consistent policy for sex education in Iran, and on the other, the predominant atmosphere in the society and the Ministry of Education prevents educators from dealing with this issue seriously. Although international organizations recognize sexual health education as a human right and a requirement for developing and propagating justice (52), there is a resistance to sex education in Iran, which was both pointed out by our participants and inferred from contemporary literature review on the subject and media. So far, it seems impossible to have an integrated and specific policy for sex education within the Iranian educational system. In 2005, the Ministry of Health and Medical Education and the Ministry of Education jointly developed and proposed for executing a comprehensive curriculum for health education in schools from pre-school to the end of college (53). The second chapter of the curriculum – titled “Social and Family Health” – offered discussions about maintaining sexual self-control, preventing teen pregnancy, and identifying unhealthy and harmful relationships. Information on sexually transmitted diseases was sporadically explained among other discussions in other chapters. However, resistance to this curriculum did not allow it to be included in the Iranian system of education. Ever since, these discussions have been offered at schools sporadically and at the discretion of those in charge, and recently under the title “Prevention of Social Harms” (53). 

Moreover, we can refer to the two courses about puberty and marriage in universities titled “Population and Family Planning” which changed to “Population and Family Knowledge” since the first semester of the 2003-2004 educational year due to changes in the national policies concerning population. This is a core course that university students of all majors must pass to get their bachelor’ degree. 

Ethical challenges that parents or teachers face in sex education are complicated (29). In order to resolve such challenges, we should decide ethically based on the four basic principles of medical ethics (autonomy, beneficence, non-maleficence, and justice) (54). To verify whether a state is ethical or not, we have to determine which principle is superior to the other ones for resolving the ethical dilemma, and then employ this principle to assess the state and make a decision (30). Seemingly, non-maleficence (one ought not to inflict evil or harm) and beneficence (one ought to do or promote well) are two significant principles that have to be taken into consideration.

***Limitations of the Study***

One limitation of the present study was that some people are ashamed to talk about this subject, although due to the scientific outlook and description of the study objectives, most of the participants answered the questions thoroughly and clearly. If the participants’ answers happened to be ambiguous, the subject would be clarified through more specific, in-depth questions. Another limitation of this study was the absence of adolescents among the participants. Participants were asked to express whether they desired to have had knowledge of these contents in their teenage years or not to overcome this limitation. 

## Conclusion

Clearly, one of the main issues relating to adolescence is teaching the various aspects of pubertal health. Not having information or having wrong information about sexual subject matters not only increase the risk for occurrence of a range of sexual disorders, high-risk behaviors, sexually transmitted diseases, unwanted pregnancies and family problems, but have harmful effects on adolescents’ lives. Embarrassment and shame, the taboo of talking about sexual topics, and common sense conventions and beliefs in Iran are the barriers to adolescents’ access to the necessary information. Stigma and shame are among the main reasons for avoiding sexual discourse, especially at a family level. 

Nevertheless, unwillingness to talk about sexual issues is not exclusive to Iran, but is also seen in countries with similar cultural backgrounds. In these countries, there is a stiff resistance to sex education for adolescents due to a wrong understanding of the nature, objectives and effects of such knowledge; as a consequence, adolescents have no access to sex education. On the other hand, the spread of mass media and high-speed information transmission in recent years have set the ground for incorrect sex information transfer. It is not easy for many people to discuss sexuality and acceptable sexual behavior with close friends or peers, or in a broader social environment. Furthermore, since these topics concern the most personal and private parts of one’s life, there is a lack of social tendency to talk about them.

Education should not be sexually stimulating and distract a student whose mind is distant from such environments. Cultural and personality differences must be taken into account, and in addition to giving general information to all adolescents, each one should be guided individually, considering his/her needs and in specific cases. The justice principle requires that all adolescents have access to appropriate and necessary information. Education should be given based on adolescents’ continuum of development, and it should be suitable for their age, cultural background and family conditions. In accordance with religious experts and Islamic scriptures, this education must be embedded in the core of sexual disciplining, which starts with giving gender identity to children and continues in every phase of their physical and mental development, depending on their needs. Considering adolescents’ age and background information at the time of sex education is highlighted indirectly. The proper time for education in different aspects of pubertal health (physical, sexual, mental, and social) is different. It is important that each element of puberty be taught to adolescents at its proper time and considering their cultural and social condition. Decision-making is complicated when adolescents’ parents are against such education. It appears that not giving the right knowledge to adolescents, who can access an ample amount of wrong information, is in contrast with principles of non-maleficence and beneficence. 

In this regard, the degree of adolescents’ understanding and capacity should serve as the criterion, and it is also very helpful to educate and persuade parents in such cases. Furthermore, confidentiality is often a key concern, and autonomy should be respected.

## Notes:

**Citation to this article:**

Joodaki K, Nedjat S, Vahid Dastjerdi M, Larijani B. Ethical considerations and challenges of sex education for adolescents in Iran: a qualitative study. J Med Ethics Hist Med. 2020; 13: 2.
